# Pathogen identification and outcome in adult patients with community-acquired pneumonia in Switzerland: findings from the Swiss CAPNETZ cohort study

**DOI:** 10.1007/s15010-025-02699-2

**Published:** 2025-11-27

**Authors:** Samuel Etienne, Werner C. Albrich, Mathias W. Pletz, Marcus Panning, Vivian Suarez Domenech, Frank Eberhardt, Grit Barten-Neiner, Daiana Stolz

**Affiliations:** 1 Clinic of Respiratory Medicine and Pulmonary Cell Research, University Hospital of Basel, Basel, Switzerland; 2 Unit for Lung and Airway Research, Institute of Environmental Medicine, Karolinska Institute, and Center for Molecular Medicine, Karolinska University Hospital, Stockholm, Sweden; 3 Division of Infectious Diseases, Infection Prevention and Travel Medicine, Cantonal Hospital, St. Gallen, Switzerland; 4 Institute of Infectious Diseases and Infection Control and Center for Sepsis Care and Control (CSCC), Jena University Hospital, Jena, Germany; 5 Medical Center and Faculty of Medicine, Institute of Virology, University of Freiburg, Freiburg, Germany; 6 CAPNETZ STIFTUNG, Hannover, Germany; 7 German Centre for Lung Research, Biomedical Research in Endstage and Obstructive Lung Disease Hannover (BREATH), DZL, Hannover, Germany; 8 Clinic of Respiratory Medicine, Medical Center and Faculty of Medicine, University of Freiburg, Freiburg, Germany

**Keywords:** Community-acquired pneumonia, Etiololgy, Streptococcus pneumoniae, Haemophilus influenzae, Immunosupression, Polymerase chain reaction

## Abstract

**Background:**

Community-acquired pneumonia (CAP) causes significant morbidity and mortality, but Swiss data are limited. We analyzed the Swiss CAPNETZ cohort to describe patient characteristics, pathogens, diagnostics, and outcomes.

**Methods:**

Adults with CAP were prospectively enrolled between 2010 and 2022 at two tertiary hospitals. Data on demographics, comorbidities, microbiology, and outcomes were collected. Mortality was assessed at 28 and 180 days.

**Results:**

Among 478 patients, 97.7% were hospitalized (median 7 days), and 76.4% had ≥ 1 comorbidity. ICU admission occurred in 7.3%. Overall mortality was 2.9% at 28 days and 5.5% at 180 days, increasing during the COVID-19 pandemic (28-day: 5.7%; 180-day: 10.9%). Higher mortality was observed in older, immunosuppressed, and oncologic patients. The etiological pathogens were detected in 38.3%, and molecular testing of sputum and bronchioalveolar lavage (BAL) enhances pathogen detection rates. Bacterial monoinfections predominated (20.2%), followed by viral (7.4%) and mixed infections (9.5%). Leading pathogens were *Streptococcus pneumoniae* (35.0%) and *Haemophilus influenzae* (16.1%). In immunocompromised patients, *H. influenzae* predominated.

**Conclusion:**

Overall mortality of CAP stays high. *S. pneumoniae* remains the most common pathogen overall, while *H. influenzae* is the most frequent pathogen in immunocompromised patients. Molecular testing of sputum and BAL improves pathogen detection.

**Supplementary Information:**

The online version contains supplementary material available at 10.1007/s15010-025-02699-2.

## Introduction

Community-acquired pneumonia (CAP) represents a significant public health challenge due to its associated mortality, morbidity, and costs [1, 2].

In European countries, the incidence of CAP has been reported to be 1.6 to 10.8 per 1000 adults annually [3]. A prospective Spanish study reported that 30-day mortality in non-immunosuppressed patients with CAP requiring hospitalization decreased from 9.6% in the 1995–1999 period to 4.1% in the 2010–2014 period, despite patients getting older and having more comorbidities [4]. For CAP patients admitted to the Intensive Care Unit (ICU) for organ support — termed "severe CAP" by some authors [5] — the 30-day mortality ranges from 27 to 33% [6, 7]. Precise data for Switzerland are unavailable, as CAP is not a notifiable disease in this country. The annual incidence of CAP requiring hospitalization was reported to be 536/100,000 inhabitants between 2011 and 2015, with 5% attributed to pneumococcal CAP (annual incidence of pneumococcal CAP 27/100,000 between 2011 and 2015) [8]. In 2001, the all-cause mortality rate for adults hospitalized with CAP in Switzerland was estimated to be 8.6% [9].

Despite comprehensive laboratory investigations, the CAP etiology identification rate varies from 36.5% to 81%. [10–13]. In the German multicenter observational CAPNETZ cohort study, patients who underwent “complete”, protocol driven diagnostics – at least urine-antigen testing for *Streptococcus pneumoniae*/*Legionella* spp, blood culture, at least one respiratory sample (sputum or BAL or tracheobronchial secretion) for conventional bacterial culture and PCR for respiratory viruses – had significantly higher pathogen detection rates compared to those who did not receive complete diagnostics (21.2% vs. 6.4%, respectively) [14]. Uematsu et al. demonstrated that adherence to guidelines for microbiological testing—encompassing sputum tests, blood cultures, and urine antigen tests conducted on the first day of hospitalization—was significantly correlated with reduced 30-day mortality rates and an increased probability of discharge alive [15].

This underscores the importance of robust guidelines, which require knowledge of pathogens, their distribution in different populations, and local antibiotic resistance rates. In this analysis, we present the results from the multicenter observational Swiss CAPNETZ cohort study, which describes patient characteristics, diagnostics, pathogen distribution, therapy, and outcomes of adult patients presenting with CAP in Switzerland.

## Patients and methods

### Swiss CAPNETZ cohort

The Swiss CAPNETZ cohort was initiated as a prospective, observational sub-study to collect data on CAP patients via the platform and eCRF of the German Competence network study of CAP (CAPNETZ, http://www.capnetz.de) with 39 sites in Europe, thereof 33 centers in Germany. Recruitment of the Swiss patients took place at two large tertiary care academic hospitals with diverse catchment areas in Switzerland between 2010 and 2022 (i.e. University Hospital Basel and Cantonal Hospital St. Gallen).

### Inclusion/exclusion criteria

Criteria for inclusion were suspected CAP in subjects ≥ 18 years, a pulmonary infiltrate diagnosed by chest radiography and/or CT scan, and at least one of the following criteria: history of fever (temperature ≥ 38.3 °C), cough, production of purulent sputum or focal chest signs on auscultation [2]. Known active tuberculosis or possible nosocomial acquisition of infection were exclusion criteria. Since April 2018, data collection included centralized viral and bacterial qualitative polymerase chain reaction (PCR) for influenza A and B viruses, respiratory syncytial virus A/B, human metapneumovirus A/B, human bocavirus, parainfluenza virus 1–4, human coronavirus HKU1, NL63, 229E and OC43, rhinovirus, enterovirus, adenovirus, SARS-CoV-2 Virus, *Mycoplasma pneumoniae*, *Chlamydia pneumoniae*, *Legionella pneumophila/longbeachae*, *Bordetella pertussis*; *Streptococcus pneumoniae*, *Staphylococcus aureus*, *Haemophilus influenzae*, *Moraxella catarrhalis* analyses performed on nasopharyngeal swabs at the central study facilities at the University Hospital of Freiburg, Germany (viral multiplex PCR) and University Hospital of Lübeck, Germany (bacterial multiplex PCR) [16]. Additionally, timing of initiation of antibiotic treatment became available. Since March 2020, the study also included patients with SARS-CoV-2 infection and implemented centralized PCR testing for SARS-CoV-2 in nasopharyngeal swabs.

Since January 2020 patients with chronic severe immunosuppression and human immunodeficiency virus infection were considered for inclusion. Chronic severe immunosuppression was defined as ongoing pharmacological immunosuppression (including chronic systemic steroid therapy) following transplantation, or due to rheumatological or other underlying diseases, chemotherapy, congenital or acquired immunodeficiency syndromes, active hematological neoplasia, or neutropenia.

All clinical and vital signs and score parameters were evaluated at first patient contact in the emergency department. Patients were followed-up according to a standardized protocol for up to 180 days and all clinical parameters were stored in a centralized, electronic database. To evaluate outcome parameters including confirmation of vital status, patients or their relatives were contacted either personally or by phone for structured interviews at 30 and 180 days. CRB-65 [Confusion of new onset, defined as an abbreviated mental test score of ≤ 8, Respiratory rate of ≥ 30 breaths/min, Blood pressure < 90 mmHg systolic or diastolic blood pressure ≤ 60 mmHg, Age ≥ 65 years] scores were calculated based on the sum of points, with one point assigned for the presence of each criterion [17]. Written informed consent was obtained from every patient before inclusion in the study, and the study was approved by the local ethical committees of each participating center (BASEC ID 2009-296).

### Criteria for definition of causative agents

A bacterial pathogen was determined to be present if it was detected in either blood samples, sputum, endotracheal aspirate, or bronchoalveolar-lavage (BAL) specimen by means of culture; if *M. pneumoniae* was detected in a nasopharyngeal or oropharyngeal swab, in sputum or BAL specimen by means of PCR assay; if *L. pneumophila* was detected in sputum by means of PCR assay; or additionally if *L. pneumophila* or *S. pneumoniae* were detected in urine by means of antigen detection. A viral pathogen was determined to be present if a PCR assay was positive in a nasopharyngeal or oropharyngeal swab, in sputum and or in bronchoalveolar-lavage specimen.

### Statistical analysis

Continuous variables were presented as medians with interquartile range. Categorical variables were presented as frequencies and percentages of the specified group. Comparisons between groups were made with the Chi-square test. A two-sided p-value of < 0.05 was considered statistically significant. Statistical analyses were performed using SPSS Statistics, version 28, software (IBM, Armonk, NY, USA).

## Results

### Patient population and outcome

A total of 489 patients were considered for eligibility May 2010 to November 2022. Eleven patients did not have an infiltrate in imaging studies and therefore did not meet inclusion criteria. Thus, 478 patients were included. The great majority of patients, 467 (97.7%), required hospitalization, with a median length of hospital stay of 7 days (interquartile range 5 to 10 days). Eighty-two (17.2%) patients had documented antibiotic therapy prior to study inclusion.

Most patients (364/478, 76.4%) had at least one documented comorbid condition, with the most common being chronic renal insufficiency (19.5%); diabetes mellitus (19.2%); as well as COPD and chronic heart failure (both 15.3%) respectively. In the subgroup recruited after the April 2018 amendment, malignant disease was noted in 32.2%. In the subgroup recruited after the March 2020 amendment, immunosuppression was noted in 19.2%.

A positive vaccination status was reported by 5.2% of patients for pneumococcal vaccine, 38.9% for seasonal influenza and, for the period after 2020, 76.4% for SARS-CoV-2. Among patients > 65 years of age, pneumococcal vaccination rate was 6.0%.

### Treatment setting

Among the 372 patients for whom the CRB65 score was available, the majority (176 patients, 47.3%) had a score of 1 point. Another 127 patients (34.1%) had a score of 0 points and therefore might have been managed ambulatory in terms of CAP severity (Table 1).

**Table 1:** Patients’ characteristics at inclusion and clinical outcomes

Characteristic	Adults with CAP (n = 478, 100%)
Median age—years (IQR)	66 (54 – 76)
Age group distribution– n (%)	
18–50 years	96 (20.1)
51–65 years	134 (28.0)
66–80 years	183 (38.3)
≥ 81 years	65 (13.6)
Female – n (%)	178 (37.2)
Inpatient treatment for CAP – n (%)	467 (97.7)
Length of hospital stay – days (IQR)^a^	7 (5–10)
Comorbidity – n/total n (%)	
any	364 (76.4)
Any chronicChronic lung disease	118 (24.7)
COPD	73 (15.3)
Asthma	34 (7.1)
Bronchiectasis	12 (2.5)
Pulmonary fibrosis	5 (1.0)
Sarcoidosis	3 (0.6)
Chronic ventilatory support	5 (1.0)
Congestive heart failure	73 (15.3)
Diabetes mellitus	92 (19.2)
Chronic renal insufficiency	93 (19.5)
Chronic liver disease	15 (3.1)
Cerebrovascular disease	27 (5.6)
Malignant disease^a^	92/285 (32.2)
Severe immunosuppression ^a^	55/285(19.2)
HIV^a^	9/285 (3.2)
Vaccination status – n (%)	
Influenza	186 (38.9)
Pneumococcal	25 (5.2)
PCV 13^b^**	9/285
PPV 23^b^**	1/285
COVID-19*	81/106 (76.4)
Antibiotic treatment within 4 weeks before inclusion – n (%)	82 (17.2)
CRB-65 score – n/total n (%) ^c^	
0	127/372 (34.1)
1	176/372 (47.3)
2	64/372 (17.2)
3	5/372 (1.3)
4	0/372 (0)
ICU admission (any time) – n (%)	
overall	35 (7.3)
Non-invasive ventilation	14 (2.9)
Mechanical ventilation with intubation	5 (1.0)

*IQR* interquartile range

^a^Information available only in patients included after the 2018 study amendment (n = 285)

^b^Missing data in 2 patients

^c^Missing data in 106 patients

A total of 35 patients (7.3%) required admission to the ICU at any time during hospitalization. Among those, 14 patients (2.9%) required non-invasive ventilation, and 5 (1.0%) required mechanical ventilation with intubation. ICU admission rate (10.4%) as well as invasive and non-invasive mechanical ventilation rates (5.2% and 1.5%, respectively) were highest in the 51–65 years age group. Among patients with comorbidities, ICU admission rate was highest among patients with chronic heart failure (9.6%).

### Outcome

Overall mortality at 28 and 180 days were 2.9% and 5.5%, respectively. None of the 11 patients managed on an outpatient bases died. In patients requiring hospitalization, mortality at 28 and 180 days were 3.0% and 5.6%, respectively. Octogenarians and patients with malignant conditions depicted the highest mortality at 30 and 180 days (6.2% and 9.2% and 8.7% and 16.3%, respectively). Similarly, admission to intensive care was associated with death (17.1% at 28 days and 25.7% at 180 days) (Table 2).

**Table 2:** major clinical outcomes among patients according to age group and comorbidities

		Mortality at 28 days	Mortality at 180 days	ICU admission		
					Non-invasive ventilation	Mechanical ventilation with intubation
Patient age groups – n/total n (%)	18–50 years	1/96 (1)	3/96 (3.1)	6/96 (6.3)	2/96 (2.1)	0/96 (0)
	51–65 years	5/134 (3.7)	8/134 (6.0)	14/134 (10.4)	7/134 (5.2)	2/134 (1.5)
	66–80 years	4/183 (2.2)	8/183 (4.4)	10/183 (5.5)	2/183 (1.1)	3/183 (1.6)
	≥ 81 years	4/65 (6.2)	6/65 (9.2)	5/65 (7.7)	3/65 (4.6)	0/65 (0)
Patients with comorbidities – n (%)	COPD (n = 73)	2 (2.7)	2 (2.7)	4 (5.5)	3 (4.1)	0 (0)
	Any pulmonary comorbidity^a^ (n = 118)	3 (2.5)	5 (4.2)	6 (5.1)	3 (1.5)	0 (0)
	Chronic heart failure (n = 73)	1 (1.4)	2 (2.7)	7 (9.6)	3 (4.1)	1 (1.4)
	Diabetes mellitus (n = 92)	3 (3.3)	3 (3.3)	7 (7.6)	4 (4.3)	1 (1.1)
	Malignant disease^b^ (n = 92)	8 (8.7)	15 (16.3)	6 (6.5)	2 (2.2)	1 (1.1)
	Severe immunosuppression^b^* (n = 55)	4 (7.3)	8 (14.5)	3 (5.5)	0 (0)	1 (1.8)
Patients admitted to ICU – n/total n (%)		6/35 (17.1)	9/35 (25.7)	N/A	14	5

^a^The group any pulmonary comorbidity includes patients with reported COPD, asthma, bronchiectasis, pulmonary fibrosis, sarcoidosis, and/or baseline chronic ventilatory support

^b^Information available only in patients included after the 2018 study amendment (n = 285)

During the COVID-19 pandemic (2020–2022), mortality was 5.7% at 28 days and 10.9% at 180 days, and thus significantly increased as compared to the precedent years (1.3% and 2.0%, respectively). Mortality rates were higher among patients with CAP with a positive SARS-CoV-2 PCR (13.3% at both 28 and 180 days) compared to those without (1.8% and 7.2%, p = 0.006 and p = 0.2381) (Table 3).

**Table 3:** Comparison of the overall mortality rates and mortality rates during the 2010–2019 period and the 2020–2022 period

	Overall	2010–2019 period	2020–2022 period		Relative risk (95% confidence interval)	p-value
28 days mortality rate	2.9%	1.3%	5.7%		0.4419 (0.1809 to 0.8495)	0.009
			SARS-CoV2 positive	SARS-CoV2 negative		
			13.3%	1.8%	1.843 (1.290 to 2.271)	0.006
180 days mortality rate	5.5%	2.0%	10.9%		0.3774 (0.1803 to 0.6862)	0.0002
			SARS-CoV2 positive	SARS-CoV2 negative		
			13.3%	7.2%	1.345 (0.8756 to 1.801)	0.2381

### Pathogen detection and pathogen distribution

**Fig. 1:**
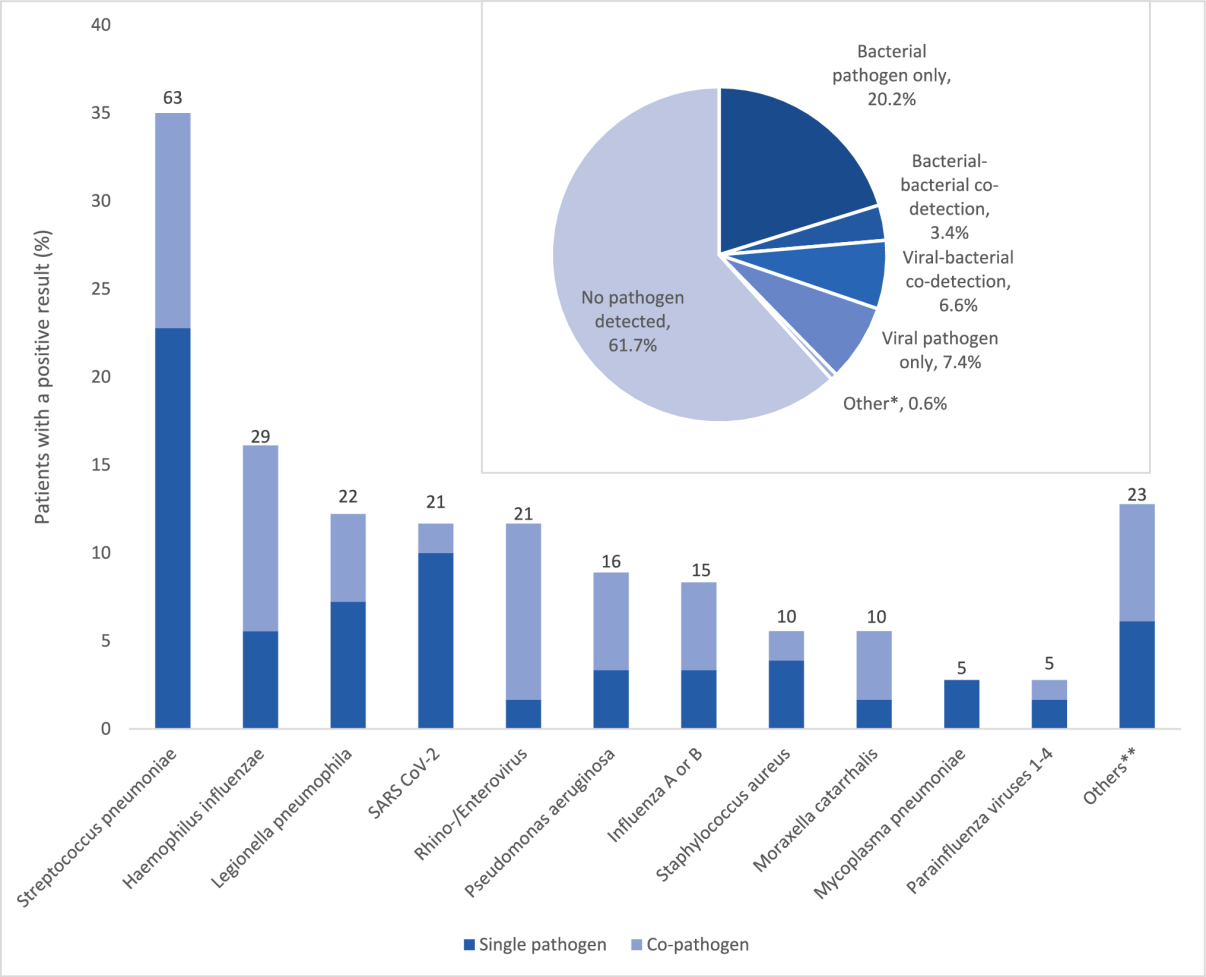
Identified pathogens. Numbers (above the bars) and percentages of patients in whom a specific pathogen was detected. Pie chart showing the proportions of bacterial, bacterial-bacterial, viral-bacterial, viral, and other pathogens detected and no pathogen detected. *Other includes: one patient with a bacterial-fungal co-infection (*E. coli, S. pneumoniae* and *Aspergillus spp*), one patient with a viral-fungal co-infection (Rhinovirus/Enterovirus and *Aspergillus fumigatus*) and one patient with a fungal infection (*Aspergillus spp*). **Others include: *E. coli* (n = 4), Enterobacteriaceae (n = 2), Coronavirus (non-SARS-CoV-2) (n = 2), *Serratia marcescens* (n = 2), Metapneumovirus (n = 2), respiratory syncytial virus (n = 2), *Aspergillus spp* (n = 2), *Aspergillus fumigatus* (n = 1), bacteria not otherwise specified (missing data) (n = 1), *Streptococcus pyogenes* (n = 1), *Klebsiellae* spp. (n = 1), *Chlamydia pneumoniae* (n = 1), *Streptococcus agalactiae* (n = 1), *Streptococci* (β-hemolytic) non-A, non-B (n = 1)

At least one sample for microbiological testing was available in 470/478 (98.3%) patients. Among these, a pathogen was detected in 180 patients (38.3%): a single bacterial pathogen was detected in 95 patients (20.2%), and a single viral pathogen in 35 patients (7.4%). Thirty-one patients (6.1%) had a viral-bacterial co-infection, and a co-infection with two bacteria was detected in 16 patients (3.4%) **(**Fig. 1A). Among patients with severe immunosuppression, a pathogen was detected in 26/55 (47.3%) patients; 5/55 (9.1%) had a single bacterial pathogen, 8/55 (14.6%) a single viral pathogen, 8/55 (14.6%) a viral-bacterial co-infection and 2/55 (3.6%) a bacterial-bacterial co-infection.

The most frequently identified pathogens were *S. pneumoniae*, detected in 63/180 patients (35.0%), *H. influenzae* in 29 (16.1%) and *L. pneumophila* in 22 patients (12.8%) (Fig. 1B).

**Fig. 2:**
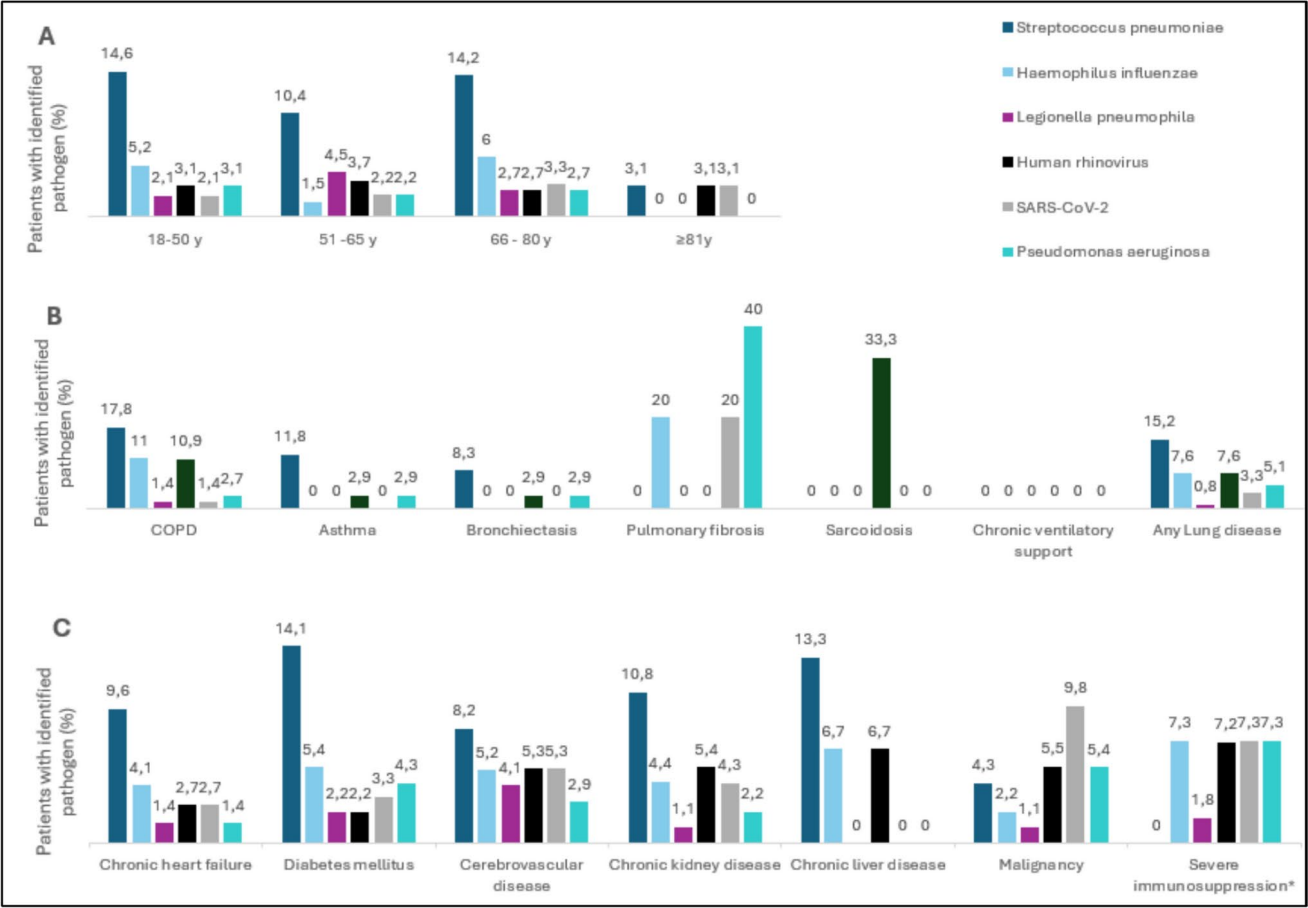
Pathogen distribution according to age and comorbidities. Distribution of the six most frequently identified pathogens according to **A** age group, **B** presence of pulmonary comorbidities, and **C** presence of other comorbidities. *Information available only for patients enrolled after the 2018 study amendment (n = 285)

*S. pneumoniae* was the most frequently identified pathogen in all age groups (ex aequo at 3.1% with Human rhinovirus and SARS-CoV-2 virus in the ≥ 81 years of age group). It was identified in 4.3% of patients with malignant disease but in none of the patients with documented severe immunosuppression. In these patient subgroups, the most frequently identified bacterial pathogens were *H. influenzae* (2.2 and 7.3%, respectively) and *P. aeruginosa* (5.4 and 7.3%, respectively) (Fig. 2).

### Diagnostic yield of microbiological tests

Blood cultures were drawn at least in one occasion from 404 patients (84.5%), a urine specimen was obtained for *S. pneumoniae* and for *L. pneumophila* antigen detection from 364 and 389 (76.1% and 81.4%) patients, respectively, a sputum specimen for culturing from 129 (27.0%) patients; and a bronchoalveolar-lavage specimen for culturing from 165 (34.5%) patients. An endotracheal aspirate for culturing was obtained from 4 of 193 patients included before 2018 (2.1%), and in no patient (0%) after the 2018 amendment. Nasopharyngeal and oropharyngeal swabs were obtained from 271 of 285 patients (95.1%); viral and bacterial multiplex PCR from BAL were performed in specimens from 144 (50.5%); and bacterial PCR from sputum specimens from 6 (1.3%) patients (Table 4).

**Table 4:** Diagnostic yield of microbial testing

Sampling method	Microbiological analysis	Patients in whom test was performed	Tests yielding positive microbiological result
Sputum	Culture – n (%)	129 (27.0)	70 (54.3)
	Sampling before beginning of antibiotic therapy*–n/total n (%)	29/285 (10.2)	20/29 (69.0)
	Sampling after beginning of antibiotic therapy*–n/total n (%)	39/285 (13.6)	15/39 (38.5)
	PCR (bacterial) – n (%)	6 (1.3)	2 (33.3)
Endotracheal aspiration°	Culture – n/total n (%)°	4/193 (2.1)	4/4 (100)
BAL	Culture – n (%)	165 (34.5)	49 (29.7)
	Sampling before beginning of antibiotic therapy–n/total n (%)*	55/285 (19.3)	14/55 (25.5)
	Sampling after beginning of antibiotic therapy – n/total n (%)*	95/285 (33.3)	32/95 (33.7)
	PCR (viral and bacterial) – n/total n (%)*	144/285 (50.5)	38/144 (26.4)
Blood	Culture – n (%)	404 (84.5)	35 (8.6)
	Sampling before beginning of antibiotic therapy–n/total n (%)*	181/285 (63.5)	14/181 (7.7)
	Sampling after beginning of antibiotic therapy – n/total n (%)*	60/285 (21.0)	4/60 (6.7)
Urine antigen test	Streptococcus pneumoniae – n (%)	364 (76.1)	36 (9.8)
	Legionella pneumophila – n (%)	389 (81.4)	12 (3.1)
Nasopharyngeal swab	Sequential centralized study PCR testing*		
	Viral – n/total n (%)*	271/285 (95.1)	63/271 (23.2)
	Bacterial – n/total n (%)*	225/285 (78.9)	98/225 (43.6)

*Information available in patients included after the 2018 study amendment only (n = 285)

°Performed in patients included before the 2018 study amendment only (n = 193); during this period no data available on timing of antibiotic initiation

A pathogen was identified in 70/129 sputum cultures (54.3%), mostly *H. influenzae* (7.0%) and *S. pneumoniae* (6.2%); in 49/165 BAL specimen cultures (29.7%), mostly *Pseudomonas aeruginosa* (4.8%) and *S. aureus* (3.6%); and in 35/404 blood cultures (8.6%), mostly *S. pneumoniae* (4.0%) and *P. aeruginosa* (0.5%).

For sputum culture (and as a trend for blood cultures), the rates of positive results were higher if sampling occurred before the initiation of antibiotic therapy (69.0% vs 38.5%, p = 0.013 and 7.7% vs. 6.7%, respectively). In contrast, for BAL culture, positive results were found in 25.7% of patients with BAL before the start of antibiotic therapy and in 33.7% of those in whom BAL occurred after the initiation of the antibiotic therapy (p = 0.359). Among participants who underwent BAL after starting antibiotics, the procedure was performed at a median of 1 day (IQR 1–2 days) following antibiotic initiation.

Viral and bacterial multiplex PCR performed in BAL specimens yielded positive results in 38/144 (26.4%) cases; most frequently identified pathogens were Rhinovirus (9.0%) and Influenza A or B virus (3.5%). Sputum PCR yielded positive results in 2/6 (33.3%), revealing *M. pneumoniae* and *L. pneumophila*, respectively. Viral PCR of nasopharyngeal or oropharyngeal swabs was positive in 63/271 (23.2%), most frequently identifying SARS-CoV-2 (8.8%) and Rhinovirus (5.9%). Bacterial PCR of nasopharyngeal or oropharyngeal swabs was positive in 98/225 (43.6%), showing most frequently *S. aureus* (28.9%) and *H. influenzae* (9.8%). Urine antigen testing for *S. pneumoniae* and for *L. pneumophila* were positive in 36/364 (9.8%) and 12/389 (3.1%), respectively (Supplementary Fig. 1).

### Outcome according to identified pathogen

Patients in whom a microbiological potential pathogen has been identified had a higher mortality than patients without pathogen identification (3.9% vs. 2.3% at 28 days, and 6.7% vs. 4.4%, at 180 days), although this trend was not statistically significant (Table 5). The highest mortality was observed at 28 days for SARS-CoV-2 (9.5%) and at 180 days for *H. influenzae* (10.3%) (Supplementary Table 1).

**Table 5:** Mortality, ICU admission and n identification

	Patients without identified pathogen – n (%)	Patients with identified pathogen – n (%)	Relative risk (95% confidence interval)	p-value
Mortality				
28 days	7/298 (2.3)	7/180 (3.9)	0.7973 (0.4259 to 1.178)	0.4036
180 days	13/298 (4.4)	12/180 (6.7)	1.294 (0.7990 to 1.843)	0.2937
ICU admission				
Overall	21/298 (7.0)	14/180 (7.8)	1.067 (0.6721 to 1.544)	0.8565
NIV	9/298 (3.0)	5/180 (2.8)	0.9469 (0.4301 to 1.653)	> 0.9999
Intubation	3/298 (1.0)	2/180 (1.1)	1.063 (0.3112 to 2.083)	> 0.9999

## Discussion

Describing community-acquired pneumonia for a specific country is important because its epidemiology, clinical presentation, and outcomes can vary significantly by region due to factors like: local pathogen prevalence, antibiotic resistance patterns, health care infrastructure and access, population demographics, environmental and lifestyle factors and vaccination policies. This study presents the most recent comprehensive overview of CAP in Switzerland. Our primary findings were: (1) mortality rates were lower in our study compared to previously reported data; (2) molecular testing of sputum and BAL improved pathogen detection; and (3) *S. pneumoniae* remained the predominant pathogen overall, while *H. influenzae* was most frequently identified in immunocompromised patients.

First, the 28-day and 180-day mortality for the overall cohort as well as for the patients who required ICU admission were lower than rates reported in other studies: a retrospective analysis of a healthcare claims database in Germany reported 30-day mortality rates ranging from 2.8% in adults under 60 years of age to 26.8% in those over 60 with severe comorbidities [18]. In a previous CAPNETZ study, the overall mortality at 30 days was 5.5% [19]. In a Spanish single-center study, the 30-day mortality was reported to be 4.1% for non-ICU CAP, and 12% among those requiring ICU admission [4]. For severe CAP, 30-day mortality rates of 27% were reported in a study from the United States, while another Spanish study documented a rate of 33% [6, 7]. Compared to these studies, our cohort was relatively young (mean age 63.1 years), yet a large majority (76.4%) of participants had at least one documented comorbidity. These comparatively low mortality rates may reflect Switzerland’s well-resourced healthcare system, characterized by universal coverage, rapid access to care, and high availability of medical and ICU resources, which facilitate early diagnosis and timely treatment [20]. A strong primary care network also supports coordinated post-discharge management. Additionally, the Swiss population, particularly in German-speaking regions, tends to have favorable health behaviors, including lower smoking and alcohol consumption and higher physical activity levels [21]. At the same time, vaccination coverage against influenza and pneumococcus remains low in Switzerland: only 14% for influenza (36% in individuals ≥ 65 years) [22] and 4.5% for pneumococcus overall (9.6% in individuals ≥ 65 years, 27.1% in individuals with immunosuppression) [23]. A major reason is that at the time of the study, in adults PCV13 was recommended for persons at risk (but not for age as an independent risk factor) [24] but not licensed for adults and therefore not reimbursed, which may partly explain these low rates. Thus, while high healthcare quality and a generally healthy population may have contributed to lower mortality, limited vaccine uptake suggests potential for further improvement in the prevention of CAP.

Mortality was significantly higher during the COVID-19 pandemic compared with the pre-pandemic period, both among patients with CAP with and without documented SARS-CoV-2 infection. Interestingly, national data from Switzerland showed that hospital mortality rates for patients hospitalized with pneumonia during 2020–2021 were only marginally higher than during 2015–2019 [25]. During the first two years of the pandemic, the incidence of hospitalizations for acute exacerbations of COPD (AECOPD) and non–COVID-19 pneumonia markedly decreased in Switzerland, likely reflecting the impact of widespread social distancing and infection control measures [25]. Although overall hospital admissions declined, those who were hospitalized tended to present with more severe disease, which may explain the modestly increased in-hospital mortality observed during this period. Switzerland experienced an excess mortality in 2020 comparable to that of other European countries, with the highest peaks during the second wave (November–December 2020) and marked regional variability [26]. The regions of Basel and St. Gallen, where participants for our cohort were recruited, were less affected than others. Comparative data between Germany and Switzerland suggest that Swiss hospitals had lower mortality and ICU admission rates for severe acute respiratory infections, potentially reflecting differences in healthcare system capacity, ICU bed availability, and hospitalization criteria [27]. These contextual factors may partly explain why mortality rates in our cohort, and in Swiss national data, appeared relatively lower than those reported from some other European cohorts. Switzerland’s healthcare system maintained relatively high ICU capacity and avoided major periods of system overload, which likely mitigated mortality. It is also important to note that immunosuppressed patients were included in our study only from January 2020 onward, and mortality was highest among patients with malignancies or severe immunosuppression. Therefore, part of the observed increase in mortality may be attributable to the inclusion of these high-risk patients. Further investigation into the interplay between SARS-CoV-2–related risks, healthcare resource pressures, and organizational changes during the pandemic was beyond the scope of this study. Nonetheless, our findings support that much of the mortality associated with CAP during the pandemic reflects underlying comorbidities and immunosuppression rather than a direct effect of healthcare system strain [28].

Second, a pathogen was identified in roughly a third of patients (38.3%), which corresponds to previously published findings [14, 29, 30]. The highest diagnostic yield for pathogen identification was achieved by sputum culture, especially if collected before the initiation of antibiotic therapy, and although information on the quality of sputum sampling is lacking in many patients. This confirms prior studies highlighting the yield of appropriate sputum culture as a neglected but affordable diagnostic tool [31–33]. Sputum culture has been shown to correlate well with BAL fluid [34, 35]. However, sputum was only performed in less than a quarter of patients in our study. Thus, we postulate that higher pathogen identification rates could be achieved by performing sputum culture more frequently. Alternatively, patients with sputum production and thus prone to providing sputum for examination might carry a higher microbiological load or simply diagnostic access to the site of infection [32, 36]. If negative, BAL, including molecular testing for respiratory viruses, likely improves etiologic diagnosis in CAP. The utility of obtaining specimens for microbiological testing is an ongoing matter of debate and is recommended in patients with severe CAP and patients with immunosuppression [2, 37]. Molecular testing of lower respiratory tract samples increases pathogen detection compared with culture (39–63% vs. 71–87%) [12, 38, 39], although PCR cannot distinguish viable organisms from residual nucleic acids [40]. Despite this limitation, molecular diagnostics markedly shorten the time to pathogen identification, enabling earlier transition from empirical to targeted therapy [39, 41] and reducing unnecessary broad-spectrum antibiotic exposure [42]. This facilitates more effective antimicrobial stewardship through timely de-escalation without compromising patient safety, with studies showing no increase in adverse events, ICU transfers, or recurrence compared with conventional management [42, 43]. Although some reports suggest shorter hospital stays with molecular-guided therapy [44, 45], pooled analyses have not confirmed a significant reduction [46]. Overall, integrating molecular testing into CAP management supports more precise and individualized empirical therapy decisions while strengthening antimicrobial stewardship.

Third, *S. pneumoniae* was the most frequently identified pathogen in our study, followed by *H. influenzae*. In contrast, two recent Scandinavian studies [12, 47] identified *Haemophilus* as the most prevalent pathogen, surpassing *S. pneumoniae*. In our cohort, this was particularly evident among patients with severe immunosuppression, where Haemophilus emerged as the predominant pathogen, while *S. pneumoniae* was not detected. Haemophilus also exhibited the second-highest mortality rate in our study, exceeded only by SARS-CoV-2. This elevated mortality may be attributed to the frequent involvement of *H. influenzae* in bacterial-viral coinfections, which are known to be associated with worse clinical outcomes [48, 49]. There is variability across studies regarding CAP mortality rates by pathogen [4, 50, 51]. However, the 2016 Global Burden of Disease study identified S. pneumoniae as the leading cause of mortality in LRTIs [52]. Introduction of pneumococcal conjugate vaccines in childhood vaccination programs has been linked to decreased case-fatality rates from invasive pneumococcal disease even in adults [53, 54]. These findings have important implications for the empirical antibiotic therapy recommendations for CAP and underscore the need for tailored approaches in immunocompromised populations.

It is important to note that antibiotic treatment prior to bronchoscopy likely reduced BAL culture sensitivity for causative pathogens. In addition, antibiotics may alter upper airway flora [55] that can be carried into the lower airways during bronchoscopy. In hospitalized patients, these effects, together with antibiotic exposure, may contribute to increased detection of Gram-negative organisms, potentially explaining the relatively high rates of *Pseudomonas aeruginosa* and *Staphylococcus aureus* identified in our cohort.

Our study has several limitations. Due to its prospective design, which required patient consent, a disproportionately larger number of hospitalized patients—and potentially less acutely ill patients—were included. Consequently, this cohort may not fully represent the entire population of community-acquired pneumonia (CAP) patients in Switzerland, where primary healthcare is robust and many CAP cases are managed by General Practitioners on an outpatient basis. Additionally, the requirement to obtain informed consent in this prospective study led to a selection bias, as previously described in other CAPNETZ studies [19]. This is reflected by relatively low CRB65 scores in patients, even those admitted to ICU. Furthermore, this most likely explains the comparatively low mortality rate in our cohort.

Moreover, CAPNETZ represents a convenience sample, as patient inclusion depended on available study staff and resources rather than systematic recruitment of all hospitalized CAP cases. Consequently, only a subset of eligible patients was enrolled. While this may limit representativeness, it reflects the real-world feasibility of long-term, resource-dependent patient recruitment across participating centers.

Finally, the cohort evolved over time with the inclusion of a second study center in 2017, adding variability but also broadening the scope of the study. Furthermore, diagnostic testing was performed at the discretion of the treating physicians rather than according to a standardized study protocol. Although this could have led to variability in pathogen detection rates, it also reflects real-world clinical practices, providing insights into typical CAP management within healthcare settings and enhancing the study's relevance to everyday clinical decision-making. Notably, 98.3% of participants had at least one specimen type available for bacterial and/or viral testing, forming a solid basis for assessing pathogen distribution even with varied testing approaches.

Focusing predominantly on hospitalized cases allowed the study to capture the types of cases that impose the most substantial burden on healthcare resources, enabling a focused analysis of critical aspects of CAP management in a hospital context. The inclusion of immunocompromised patients and extensive molecular workups broadened the scope and strengthened the generalizability of the findings, providing insights from a diverse population and reflecting local pathogen profiles and resistance patterns.

In conclusion, although lower than in other countries, mortality rates of CAP remain high in Switzerland. Pathogen identification rates are low, yet molecular testing of sputum and BAL seems to enhance pathogen detection in hospitalized patients with CAP. *Haemophilus influenzae* emerged as the most frequently detected pathogen in immunocompromised patients, a finding that should be taken into consideration when developing therapy guidelines for this population.

## Supplementary Information

Below is the link to the electronic supplementary material.

Supplementary file1 (PDF 701 KB)

## Data Availability

No datasets were generated or analysed during the current study.
