# Supplementary material for: Pathogen identification and outcome in adult patients with community-acquired pneumonia in Switzerland: findings from the Swiss CAPNETZ cohort study
Source: Infection. 2025 Nov 27;54(2):717–28. doi: 10.1007/s15010-025-02699-2 (PMC13021710; doi:10.1007/s15010-025-02699-2)
Supplement: Supplementary file 1 — Supplementary file1 (PDF 701 KB) [file 15010_2025_2699_MOESM1_ESM.pdf]

Supplementary material

Pathogen identification and outcome in adult patients with community-acquired pneumonia in Switzerland: findings from the Swiss CAPNETZ cohort study

Authors and affiliations

Samuel Etienne[1,2] *, Werner C. Albrich[3] *, Mathias W. Pletz[4], Marcus Panning[5], Vivian Suarez Domenech[1], Frank Eberhardt[6], Grit Barten-Neiner[6,7], Daiana Stolz[8], for the CAPNETZ study group

[1]Clinic of Respiratory Medicine and Pulmonary Cell Research, University Hospital of Basel, Basel, Switzerland

[2]Unit for Lung & Airway Research, Institute of Environmental Medicine, Karolinska Institute, and Center for Molecular Medicine, Karolinska University Hospital, Stockholm, Sweden

[3] Division of Infectious Diseases, Infection Prevention and Travel Medicine, Cantonal Hospital St. Gallen, St. Gallen, Switzerland

[4] Institute of Infectious Diseases and Infection Control and Center for Sepsis Care and Control (CSCC), Jena University Hospital, Jena, Germany

[5]Institute of Virology, Medical Center and Faculty of Medicine, University of Freiburg, Freiburg, Germany

[6]CAPNETZ STIFTUNG, Hannover, Germany

[7]German Centre for Lung Research, DZL, Biomedical Research in Endstage and Obstructive Lung Disease Hannover (BREATH), Hannover, Germany

[8]Clinic of Respiratory Medicine, Medical Center and Faculty of Medicine, University of Freiburg, Freiburg, Germany

*These authors contributed equally to this work.

Corresponding author

Samuel Etienne, MD, email : samuel.etienne@ki.se

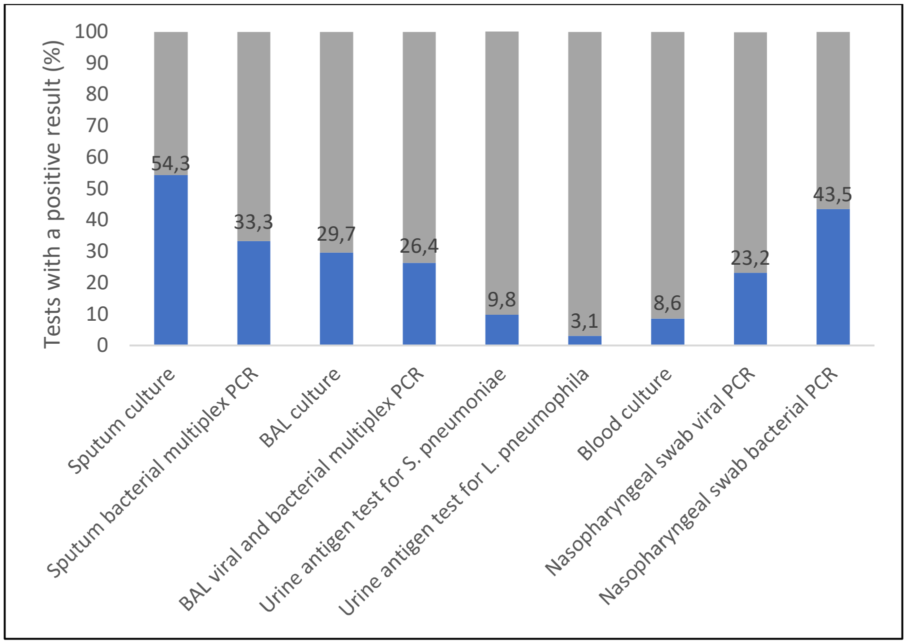

Supplementary fig. 1 Proportions of microbiological tests with positive results.

	Bacterial pathogen only	*Bacterial-bacterial co-infection*	*Bacterial-viral co-infection*	*Viral pathogen only*	*Other**	*S. pneumoniae*	*H. influenzae*	*L. pneumophila*	*Human rhinovirus*	*SARS-CoV-2*	*Pseudomonas aeruginosa*
**Mortality**											
28 days	3/95 (3.2)	0/16 (0)	0/31 (0)	2/35 (5.7)	2/3 (66.6)	1/63 (1.6)	2/29 (6.9)	0/22 (0)	0/21 (0)	2/21 (9.5)	0/16 (0)
180 days	6/95 (6.3)	1/16 (6.3)	0/31 (0)	3/35 (8.6)	2/3 (66.6)	2/63 (3.2)	3/29 (10.3)	0/22 (0)	0/21 (0)	2/21 (9.5)	1/16 (6.3)
**ICU admission**											
Overall	7/95 (7.4)	2/16 /12.5)	1/31 (3.2)	2/35 (5.7)	2/3 (66.6)	7/63 (11.1)	3/29 (10.3)	2/22 (9.1)	0/21 (0)	1/21 (4.8)	0/16 (0)
NIV	3/95 (3.2)	0/16 (0)	1/31 (3.2)	1/35 (2.9)	0/3 (0)	2/63 (3.2)	1/29 (3.4)	1/22 (4.5)	1/21 (4.8)	0/21 (0)	0/16 (0)
Intubation	0/95 (0)	0/16 (0)	0/31 (0)	1/35 (2.9)	1/3 (33.3)	1/63 (1.6)	0/29 (0)	0/22 (0)	0/21 (0)	1/21 (4.8)	0/16 (0)

Supplementary table 1 Mortality, ICU admission and mechanical ventilation rates according to identified pathogen. * Other includes: one patient with a bacterial-fungal co-infection (*E. coli, S. pneumoniae* and Aspergillus spp), one patient with a viral-fungal co-infection (Rhinovirus/Enterovirus and *Aspergillus fumigatus*) and one patient with a fungal infection (*Aspergillus spp).*
